# Electronic health record data to develop indicators of end-of-life care quality

**DOI:** 10.1136/spcare-2025-005695

**Published:** 2026-04-14

**Authors:** Stuti Bagri, Sophie Julian, Miranda Davies, Sarah Scobie, Andrea L Schaffer, The OpenSAFELY Collaborative, Amelia Green

**Affiliations:** 1 Nuffield Trust, London, UK; 2 Nuffield Department of Primary Care Health Sciences, University of Oxford, Bennett Institute for Applied Data Science, Oxford, UK; 3 TPP, Leeds, UK

**Keywords:** Advance Care Planning, COVID-19, Quality of life, Terminal Care, Palliative Care

## Abstract

**Objectives:**

An understated disruption to health services from the COVID-19 pandemic was the increase in deaths occurring outside a hospital. Measures to monitor end-of-life care focused on community settings are needed. In this study, we explore the recording and face validity of patient-centric measures of care quality at the end of life which could be derived from primary care electronic records.

**Methods:**

With the approval of National Health Service England, we analysed the OpenSAFELY-TPP electronic healthcare records of over 970 000 patients who died between March 2019 and August 2023, covering periods before, during and after the COVID-19 pandemic. After reviewing potential measures, we developed two new measures of end-of-life care provision (specialist palliative care team contacts and advance care planning) and tracked the proportion of patients with these records, categorised by place and cause of death, along with an existing measure indicating palliative care needs.

**Results:**

The proportion of people with a general practice (GP) record of specialist palliative care was 4%–5% on average and was higher for those who died of cancer or died in a hospice. Advance care planning records increased from 19% to 27%, driven in large part by increases for patients who died in care homes.

**Conclusions:**

Advance care planning and recording of palliative care needs were plausible measures to track changes in care, unlike the specialist palliative care measure, where sparse recorded use rendered it ineffectual for service monitoring. Improved coding in primary care records would enhance the reliability of measures.

**WHAT IS ALREADY KNOWN ON THIS TOPIC**

- The quality of end-of-life care is traditionally measured by how patients use health services in hospitals (eg, emergency department attendances).

**WHAT THIS STUDY ADDS**

- This study examined the scope to use general practice (GP) electronic records to develop measures of quality of end-of-life care in community settings and discussed the impact of the COVID-19 pandemic and coding incentives on trends for these measures.
- We created new measures of advance care planning and specialist palliative care to track end-of-life care delivered in the community.

**HOW THIS STUDY MIGHT AFFECT RESEARCH, PRACTICE OR POLICY**

- While the advance care planning measure showed value in understanding trends in end-of-life care, the specialist palliative care measure was sparsely coded and unlikely to be useful unless coding and data linkage between GPs and other systems improves.

## Introduction

 Life expectancy in the UK has increased over the last four decades,^1^ while the number of people living with multiple long-term conditions has increased, with 68% of adults aged 80 years or older having two or more long-term conditions.^2^ Collectively, this creates an increase in demand for end-of-life care services while simultaneously making such care more complex to deliver. While there is a large body of evidence regarding what patients value, such as sufficient pain relief, there can be a gap between patient experience and reality.^3^

The COVID-19 pandemic disrupted healthcare services globally, and the place where people died changed significantly in the UK. A third more people died at home during 2020–2022 than in the pre-pandemic (2015–2019) 5-year average, and the proportion of home deaths has since remained above pre-pandemic levels.^4^

We established a project to evaluate the changes in end-of-life care and to describe trends in where people were dying. We found significant changes in care for people who died at home,^4^ in care homes (homes for nursing, convalescence, respite and rest)^5^ and in the use of hospital services.^6^ This work was in line with WHO recommendations for COVID-19 to rapidly assess healthcare delivery and develop key performance indicators to quantitatively evaluate care.^7^

We used previously published indicators for our initial work;^8^ however, due to the substantial changes in clinical care and rapid deployment of technology,^9^ we identified a need to assess whether new indicators could be developed to support monitoring the ongoing impact of the COVID-19 pandemic in line with developments in general practice (GP) data.

Changes in place of death during the pandemic highlighted gaps in existing routine data on end-of-life care, which has been focused on hospital care and measures of activity, for example, emergency admissions at the end of life.^10^ Patient-centred outcomes are not yet routinely used when evaluating the quality of end-of-life care.^11^ With the reduction in the proportion of deaths occurring in hospitals persisting post-pandemic, routine monitoring of end-of-life care quality in primary care is essential to ensure that patients are receiving the care they need and to understand the implications for primary and community service capacity.

In this paper, we describe current indicators for measuring end-of-life care, investigate patterns of recording end-of-life care in GP electronic records and propose and assess new measures of care using linked GP, hospital and death registration data. We consider the influence of COVID-19 on the trends identified by these new measures.

## Methods

### Developing measures

To develop measures of quality of care, we reviewed literature to identify dimensions of good-quality end-of-life care.

These included effective symptom management, such as effective pain relief,^12^ coordination of services and good communication between patients, their family members and healthcare providers.^13^ A lack of integration between services is a barrier to good quality care.^14^

Receiving care from healthcare staff with specific expertise in palliative and end-of-life care is valued by patients and families, as specialist palliative care team contacts can have an important role in pain management.^15^ Continuity of contact with the specialist palliative care team is also vital in order to build trusted relationships.^16^

At its core, good-quality end-of-life care requires consulting patients on what matters to them^17^ and giving them autonomy in choosing how they are being looked after, for which a precondition is recognition that someone is approaching the end of life.^11^ Advance care planning refers to the process by which patients can state preferences about their treatment, enabling their wishes to be respected if they lose decision-making capacity.

Based on these themes, we identified six potential measures of good-quality end-of-life care: (a) recognition of end-of-life, (b) advance care planning, (c) continuity of care, (d) coordinated services, (e) specialist palliative care team contacts and (f) anticipatory medicine. To determine the feasibility of creating measures, we curated a preliminary list of corresponding Systematised Nomenclature of Medicine (SNOMED) codes, which are used by GPs in patients’ electronic health records, using data published by the National Health Service (NHS) Digital^18^ to identify key codes relating to each measure and recorded use of these codes between August 2021 and July 2022. Using this information, we consulted with our advisory group on which measures could be feasibly developed and the face validity of the measures.

For measures considered feasible, we then curated a comprehensive codelist, including all SNOMED codes that we deemed relevant to each measure. Codelists were developed using OpenCodelists, a tool that allows researchers to build their own set of SNOMED codes for use in OpenSAFELY projects. The full code lists can be found in the appendix (see online supplemental file 1) and are published on GitHub.

OpenSAFELY is a platform which enables the analysis of GP electronic health records in England. These records are digital logs of interactions and actions related to patients in a primary care setting. They can include things like medication prescriptions and clinical decisions, which are coded (using SNOMED clinical terms). However, since end-of-life care is usually delivered by providers in different settings, such as hospitals and hospices, GP records may not be a complete record of all end-of-life care received and information that is free text is not accessible.

### Data

For the three measures examined in detail, we used the OpenSAFELY data platform^19^ to explore the health service use among patients registered with a GP, using TPP (a widely used software in primary care), who died between March 2019 and August 2023. Primary care records managed by TPP were linked to hospital activity and Office for National Statistics (ONS) death data through OpenSAFELY. March 2019 was selected as the start of the period based on when information regarding the date of death was made available by the ONS within OpenSAFELY as part of our earlier study. Data management was performed using Python, with analysis carried out using R.

All data were linked, stored and analysed securely using the OpenSAFELY platform, https://www.opensafely.org/, as part of the NHS England OpenSAFELY COVID-19 service. Data included pseudonymised data such as coded diagnoses, medications and physiological parameters. No free text data are included. All code is shared openly for review and reuse under the MIT open licence. Detailed pseudonymised patient data are potentially re-identifiable and therefore not shared. More information is available in the appendix (see online supplemental file 2), along with evidence of this study’s classification as a service evaluation (see online supplemental file 3).

### Study population

Participants included patients registered with a TPP practice in England who died. Patients whose death was not registered by ONS and had invalid/missing values for sex were excluded. The TPP system covers the records of approximately 24 million patients across England. The dataset includes 975 125 people who died, which equates to 40% of all ONS-published deaths in England across the study’s period.^20^

### Data analysis

We tracked (a) the proportion of patients who died with palliative care recorded in their GP records, (b) the proportion of patients who died with specialist palliative care recorded in their GP records and (c) the proportion of patients who died with an advance care plan recorded in their GP records, in the 90 days prior to the date of death.

Although end-of-life care can be provided over a longer period, 90-days was deemed sufficient to capture the majority of end-of-life care activity and is also used by the Office of Health Improvement and Disparities to report on emergency admissions in the end-of-life period.

Measures were further subcategorised by place and cause of death. Place of death records whether patients die at ‘Home’, ‘Hospice’, ‘Hospital’, ‘Care home’ or ‘Elsewhere/other’. Cause of death was categorised as ‘Cancer’, ‘Circulatory diseases’, ‘COVID-19’, ‘Dementia and Alzheimer’s disease’, ‘Flu and pneumonia’, ‘Other respiratory diseases’ or ‘All other causes’. Developing and tracking these measures in this way allowed us to establish their validity by comparing them to what we would expect to observe in practice, supported by clinical and patient input from our advisory group.

In compliance with disclosure control policy mandated by OpenSAFELY, counts below eight were redacted and all other numbers were rounded to the nearest five. All percentages have been rounded to the nearest whole number.

### Patient and public involvement and clinical advice

The research was supported by an advisory group, including general practitioners and academic support. The advisory group provided feedback on the project plan, the quality-of-care measures selected and interpreting results.

## Results

The reasoning for our selection of measures is described in table 1.

**Table 1:** Measures considered during scoping and reasons for inclusion/exclusion in the analysis

Measure	Literature findings	Rationale for inclusion/exclusion during scoping
Included measures		
*Patients with end-of-life care (palliative care)*: codes relating to the provision of palliative care using the GP contract QOF codelist	Good-quality end-of-life care necessitates identification of patients approaching the end of life.^11^	Used in an earlier phase of the project as an indicator for people in receipt of, or identified as needing, palliative care at the end of life, inclusive of both specialist and generalist palliative care.^12^ Uses a codelist with 72 SNOMED codes predefined by NHS England.^27^
*Specialist palliative care team contacts*: a patient’s contact with members of the specialist palliative care team	Significant reduction in emergency admissions when patients were seen by the specialist palliative care team.^28^ Specialist palliative care teams have an important role in symptom control, especially around pain management.^15^ Provided by multidisciplinary teams and used when end-of-life care cannot be provided by generalist services.^20^	An adequate number of SNOMED codes relating to this measure were identified in the initial search, with eight codes and a total usage of 92 720. Following further investigation, the final codelist contained 51 codes, including codes which referred to ‘specialist palliative’, such as referral to, being seen by, under the care of or discharged from a specialist palliative care team. The advisory group noted that numbers for usage of some of the key codes included in the preliminary codelist were smaller than anticipated. However, the group suggested that, collectively, the codes could be sufficient in number.
*Advance care plan*: having a plan in place that records patients’ wishes in the event of a loss of decision-making capacity, following discussion with the patient and family^28^	Good quality care is respectful of people’s preferences, priorities and needs.^29^ Enables patients’ wishes to be respected in the event of discordance of views between patients and carers.^30^	An adequate number of SNOMED codes relating to this measure were in the initial search, with two codes and a total usage of 215 690. Following further investigation, the final codelist included 389, identified using the search terms ‘advance care plan’, ‘care plan’, ‘end of life’, ‘palliative’ and ‘treatment escalation plan’. Examples of terms include those relating to having an advanced care plan specifically for end-of-life/palliative purposes, education/discussion about care planning, agreement regarding care plans, offers for review of care plans, involvement of caregivers in care planning, clinical management plans and self-management plans for conditions that are life-limiting. The advisory group noted that the numbers for usage of these codes appeared to be under-reported based on their experiences in practice but still found it worthwhile to investigate use by cause and place of death.
Excluded measures		
*Continuity of care*: patients and family having access to the same GP throughout end-of-life care	The loss of continuity of familiar health professionals negatively impacted the advance care plan.^31^ Continuity of care with a GP was negatively associated with hospital admissions.^32^ Continuity of care was most relevant for the older population, those with comorbidities and those not living in care homes.^32^	Limited usage of relevant codes; the initial search found six relevant codes with a usage of 55 770. Tried to explore using OpenSAFELY to develop this measure in other ways beyond using SNOMED codes, but the data/resources to be able to do this are not on the platform yet.
*Co-ordination of services*: co-ordination between services, such as GP, ambulance and out-of-hours care	The importance of 24/7 shared patient records is important for both services and for patients and their families.^13^ High-quality co-ordination of services allowed for more appropriate care.^13^ Integration of services is lacking for patients.^14^	Limited usage of relevant codes; the initial search found five relevant codes with total usage of 59 220. EPaCCS has been introduced to support the co-ordination and delivery of care in accordance with patient preferences,^33^ but we cannot capture EPaCCS within the data set we used.
*Anticipatory medication*: allows access to medication patients may need if they experience an increase in the severity of symptoms or develop more symptoms while at home	Help families engage in symptom management.^34^ Could prevent emergency hospital admissions.^35^ Having a medicine kit at home will mitigate problems with delays in access to medication.^17^	Limited codes relating specifically to anticipatory medication, the initial search found two relevant codes with total usage of 42 290. A pre-existing measure relating to end-of-life medication prescribed for symptom management could better answer this question.

EPaCCS, Electronic Palliative Care Co-ordination Systems; GP, general practice; NHS, National Health Service; QOF, Quality and Outcomes Framework; SNOMED, Systematised Nomenclature of Medicine.

### Place and cause of death

Deaths most frequently occurred in hospitals (43%), followed by homes (28%), care homes (22%), hospices (5%) and elsewhere/other (3%). Cancer was listed as the main cause of death for 26% of all deaths that occurred, followed by circulatory diseases (24%), ‘other causes’ (22%), dementia and Alzheimer’s (11%), respiratory diseases (7%), COVID-19 (6%) and lastly, flu and pneumonia (4%). The profile of place and cause of death is similar to all deaths in England.^20^ The sum of deaths across all causes (n=9 75 110) is slightly lower than the study population (n=9 75 125) due to rounding of values for the purpose of disclosure control.

### Patients with end-of-life care (palliative care)

The proportion of people who died with palliative care recorded in their GP record rose from 23% to 28% across the timeframe. There was a drop in the proportion of patients who had palliative care recorded during the first wave of the COVID-19 pandemic (defined as March 2020 to May 2020), particularly for those who died in a care home (47%–41%) (figure 1). Similarly, at the start of the second wave of the COVID-19 pandemic (defined as September 2020 to April 2021), the proportion of patients who had palliative care recorded decreased, with the most notable decrease observed in those who died in hospices.

**Figure 1:**
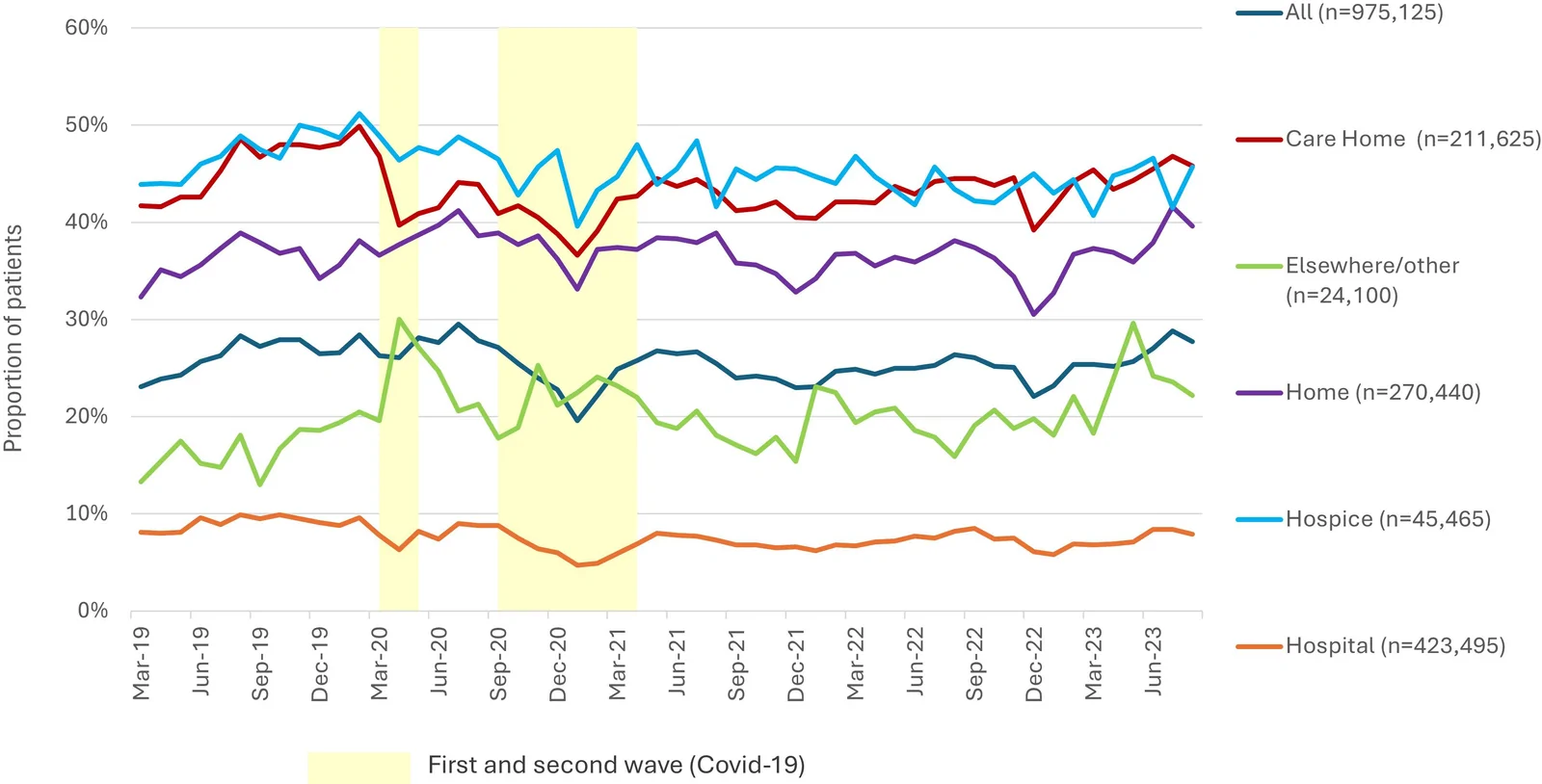
Proportion of patients who died with a record of palliative care in their general practice records in the last 3 months of life, by place of death (March 2019–August 2023).

Patients whose underlying cause of death was cancer had the largest proportion of palliative care recorded in their GP records at 45% in August 2023, followed by patients with dementia (39% in August 2023) (figure 2). For patients who died due to other conditions, the proportion with palliative care records remained below 20% for most months.

**Figure 2:**
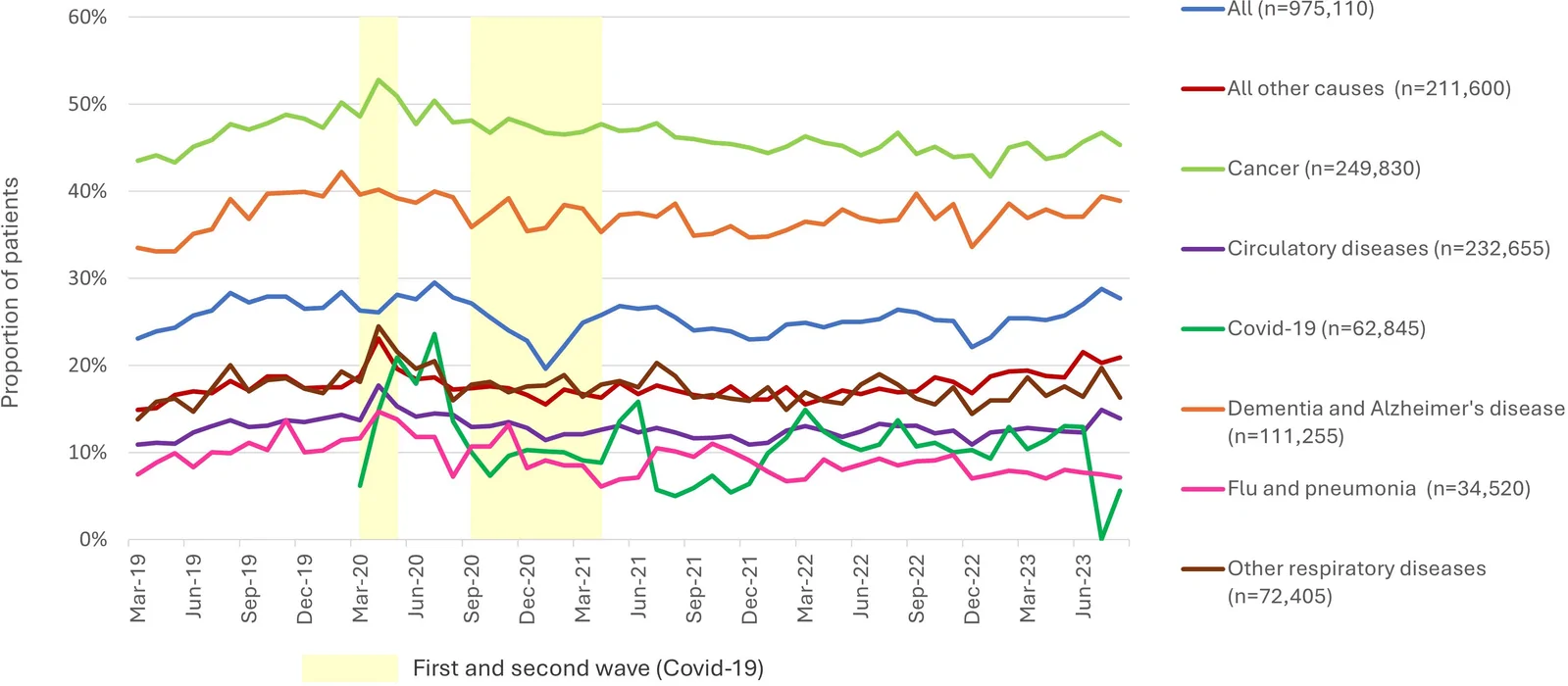
Proportion of patients who died with a record of palliative care in their general practice records in the last 3 months of life, by cause of death (March 2019–August 2023).

### Specialist palliative care

The proportion of all people who died with specialist palliative care recorded in their GP records was relatively low across the period, ranging from 4% to 5% between March 2019 and August 2023. Following the first wave of the pandemic, there was a slight increase in the number of people who had specialist palliative care recorded in their GP records.

Patients who died in a hospice consistently had the largest proportion of specialist palliative care contacts in their GP records, reaching 15% in August 2023 (figure 3). They were followed by patients who died at home, where the proportion of specialist palliative care contacts ranged from 7% to 9% across the study period.

**Figure 3:**
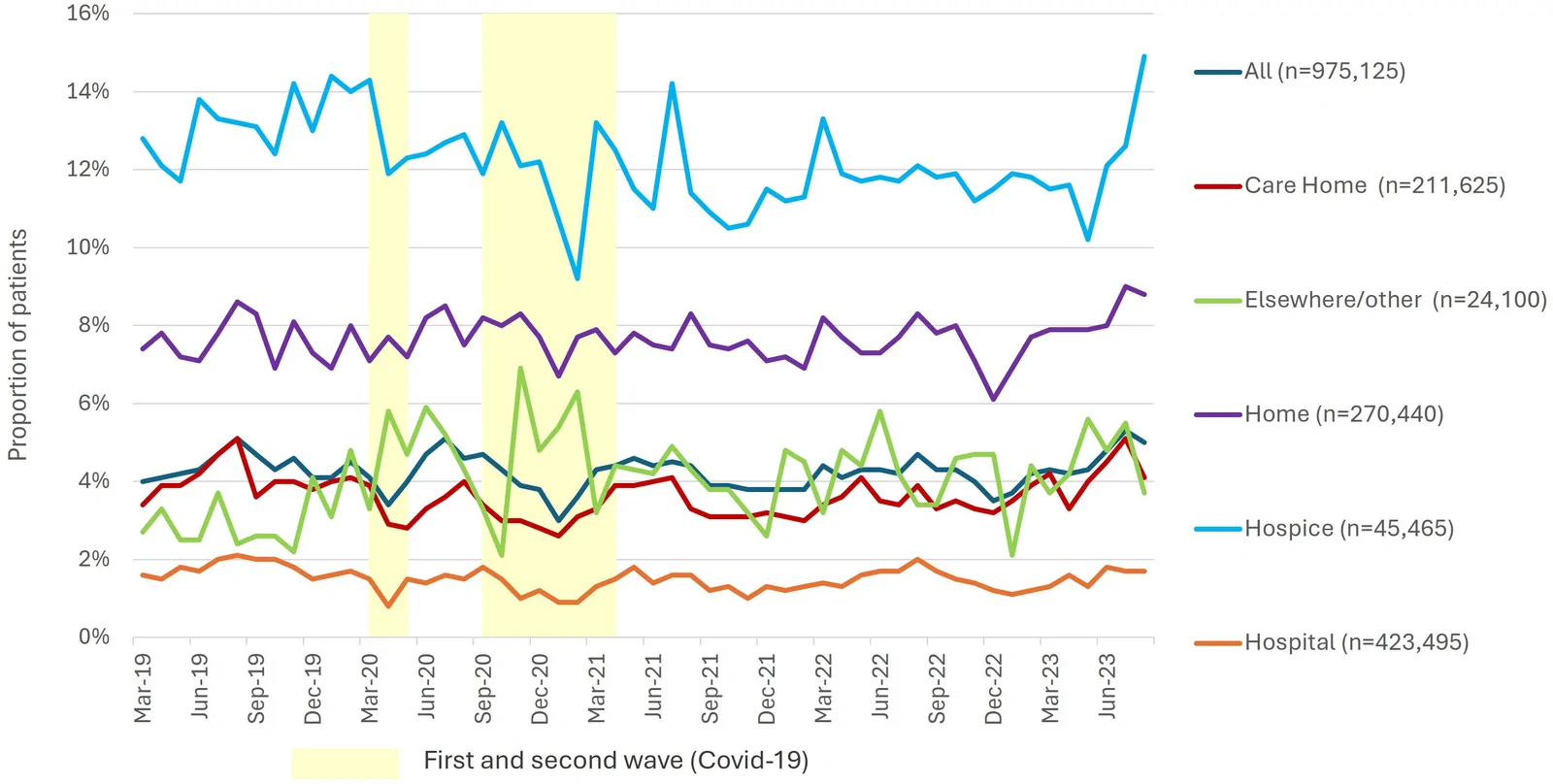
Proportion of patients who died with a record of specialist palliative care team contacts in their general practice record in the last 3 months of life, by place of death (March 2019–August 2023).

People with an underlying cause of death of cancer had the largest proportion of specialist palliative care recorded in their GP records, remaining at around 10% across the timeframe (figure 4). For all other causes of death, this mostly remained below 5% of patients. Flu and pneumonia are not shown due to small numbers of deaths, requiring disclosure redaction.

**Figure 4:**
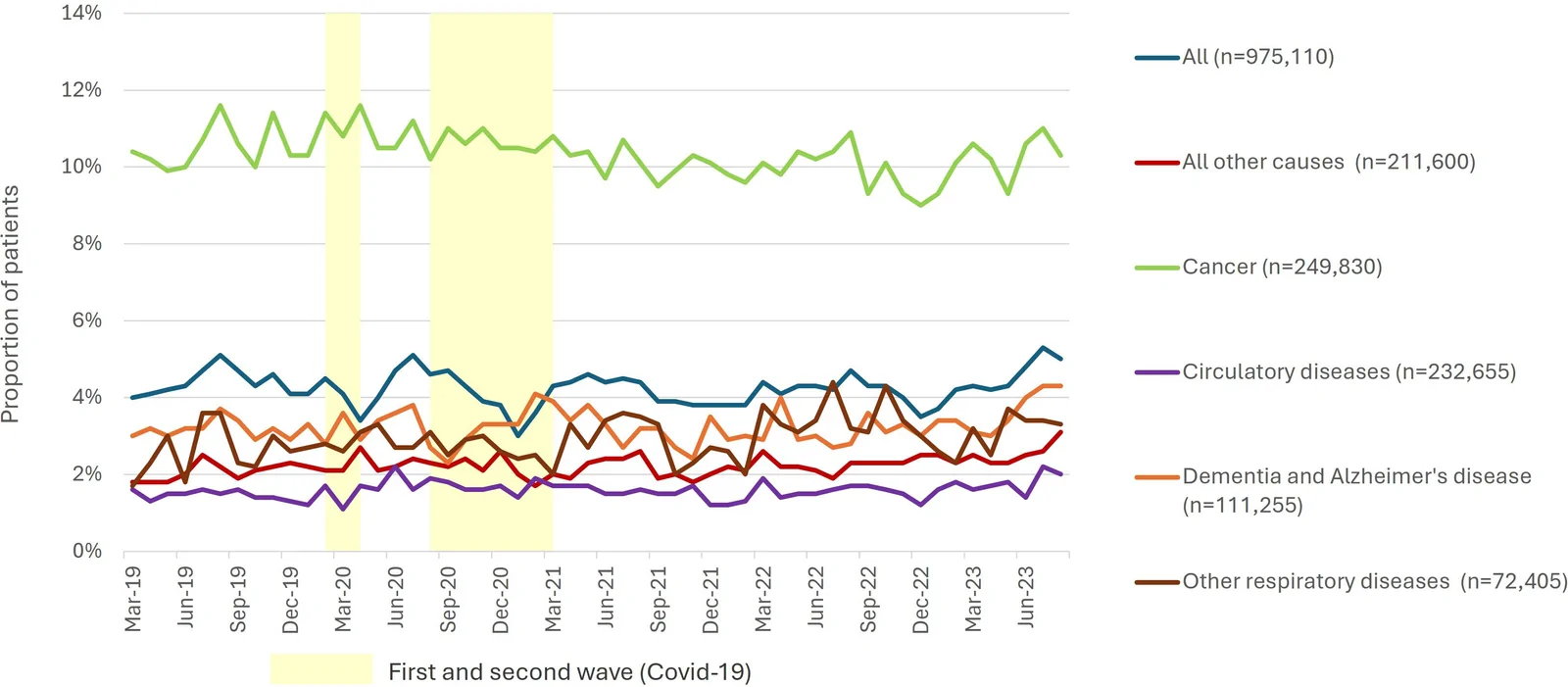
Proportion of patients who died with a record of specialist palliative care team contacts in their general practice record in the last 3 months of life, by cause of death (March 2019–August 2023).

### Advance care planning

The proportion of all people who died with advance care planning recorded in their GP records has increased over time from 19% in March 2019 to 27% in August 2023, but this was driven largely by recording specific to patients who died in care homes (figure 5). During the first wave of the pandemic, advance care planning records decreased before picking up again in early 2021. People who died in a care home consistently had the largest proportion of advance care planning records (51% in August 2023). Increases over time were far less pronounced for other groups, particularly for those who died in hospital, who also had the lowest proportion of advance care planning records across most of the study period (13% in August 2023).

**Figure 5:**
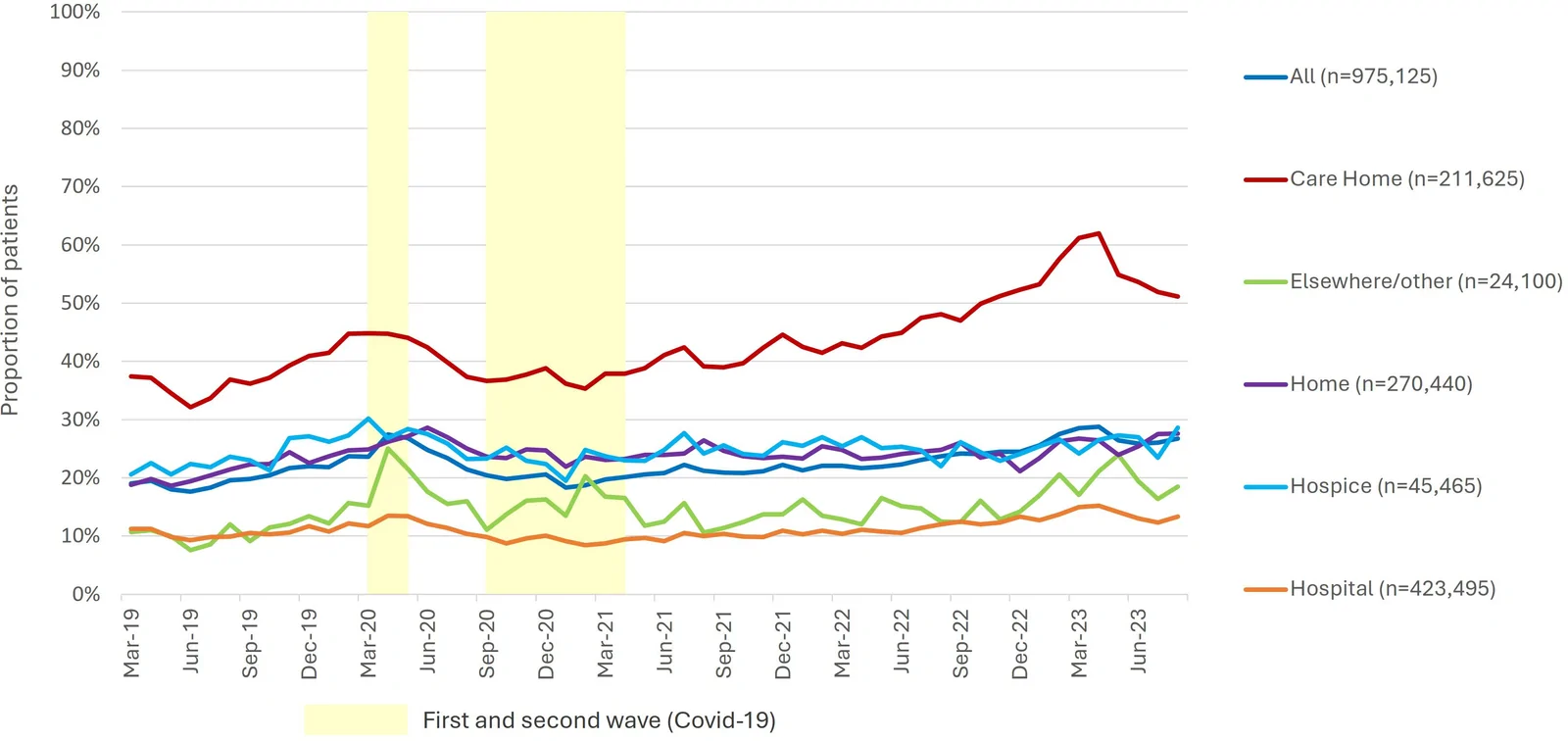
Proportion of patients who died with a record of an advance care plan in their general practice record in the last 3 months of life, by place of death (March 2019–August 2023).

People with an underlying cause of death of dementia or Alzheimer’s disease had the largest proportion of specialist palliative care recorded in their GP records, spanning 42%–47% across the study period (figure 6). For all other causes of death, this mostly remained below 30% of patients.

**Figure 6:**
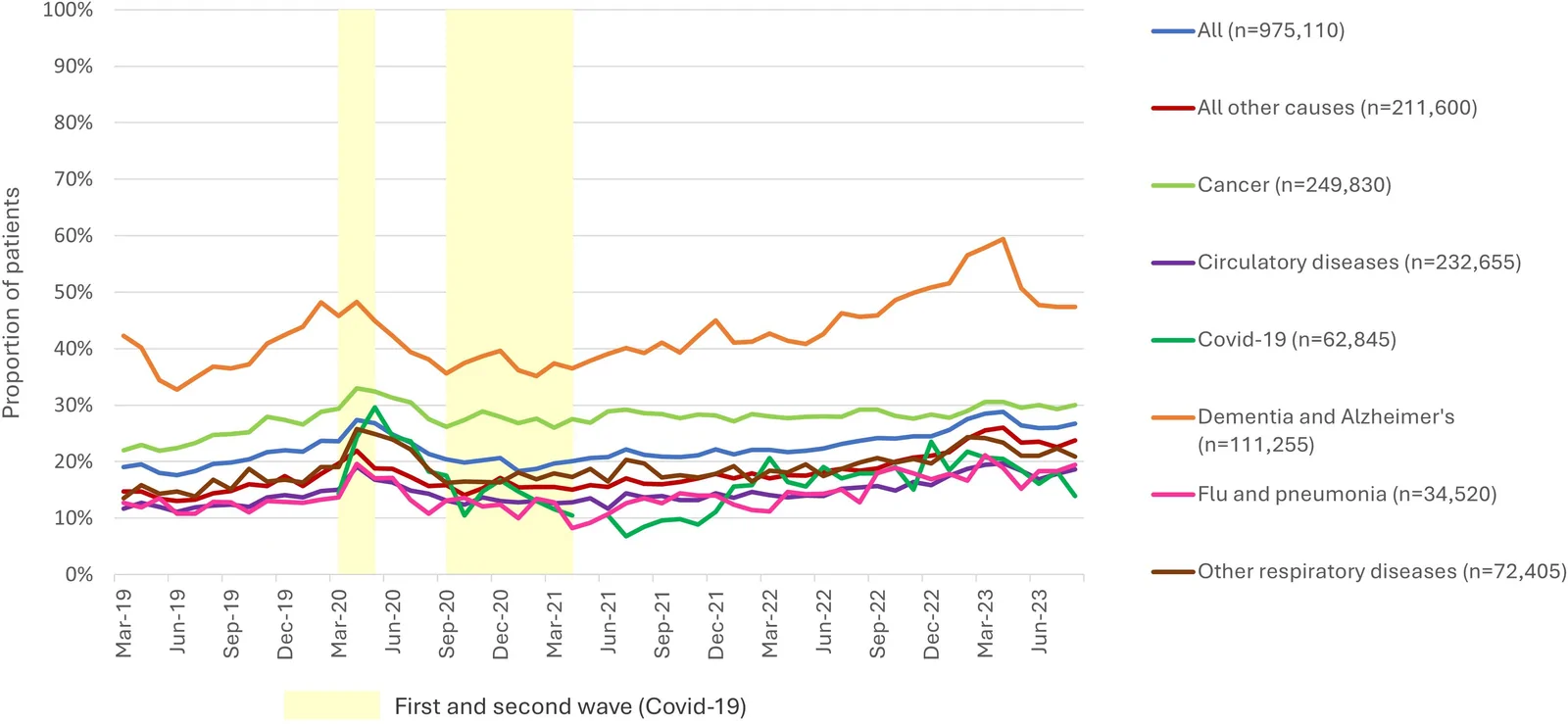
Proportion of patients who died with a record of an advance care plan in their general practice record in the last 3 months of life, by cause of death (March 2019–August 2023).

## Discussion

This study examined the scope of using GP electronic records to develop measures of quality of end-of-life care as part of a larger project to track changes in care quality resulting from the COVID-19 pandemic. The pandemic highlighted the need for measures of quality relating to care outside hospitals, with more people dying at home and a shift towards care in the community. Among a longer list of potential measures, we identified three measures to analyse in detail.

### Palliative care and specialist palliative care measures

The COVID-19 pandemic influenced the proportion of patients with palliative care recorded in their GP records. Namely, the recording of palliative care decreased following the first wave (March–May 2020) before levelling out and decreasing again during the second wave (September 2020–April 2021). There was minimal effect on specialist palliative care, likely influenced by the small proportions in this measure. Across all causes and places of death, the proportion of all patients with GP-recorded specialist palliative care contacts was consistently low, under 5% for most months. It is likely that this reflects clinical coding practice in GP records rather than the delivery of care. Feedback from the advisory group stated that most GPs group all palliative care, both generalist and specialist, together using only palliative care codes in practice. This is in line with the results from the palliative care measure, where recorded use was much higher. Overall, under current coding behaviour, the specialist palliative care measure is not a valid indicator of quality of end-of-life care. The previously developed palliative care measure had greater face validity based on volumes of data, trends and variation.

The underestimation of specialised palliative care services will be more pronounced in certain settings where primary care interaction is low. Patients who die in hospitals may be receiving inpatient specialist palliative care which would be recorded in their hospital record but likely not be reflected in their GP record. Notably, hospital deaths included a large proportion of COVID-19 deaths, but the recording of specialist palliative care codes was too small to report.

Our findings highlight some of what is already known about specialist palliative care services. Specialist palliative care is often provided locally by hospice and hospice-at-home services, and this is reflected in our findings, which show that the proportion of patients who died in hospices and at home who have specialist palliative care recorded is higher than in other settings. Additionally, the long-standing pattern of end-of-life care services being predominantly focused on providing care for patients with cancer^21^ was visible in the data, with patients who died of cancer most likely to receive specialist palliative care services compared with other causes of death.

### Advance care planning

The overall increase in the proportion of patients with a GP-recorded advance care plan was in large part driven by an increase in the proportion of patients who died in care homes with a record of an advanced care plan up until April 2023. This period coincided with the enhanced health-in-care homes programme^22^ set up to integrate care between primary care networks and care homes, which incentivised recording personalised care plans up until April 2023, after which coding dropped off. The trajectory of the proportion of patients in a care home with a GP-recorded advanced care plan matches that of patients whose main cause of death was dementia and Alzheimer’s. These patients were more likely than those who died of other clinical conditions to have a record of advance care planning, possibly because they were dying in care homes where at least a third of the residents suffer from dementia.^23^ There was an overall decrease in advance care planning records following the onset of the COVID-19 pandemic in March 2020, up until the end of the year, potentially because care provision was disrupted during that time. Since the end of 2020, the proportion of patients with advance care planning records has been increasing.

Although the usefulness of the measure will be hampered by its reliance on record sharing between GP practices and other services, the proportion of patients with an advance care plan recorded is plausible. Small increases in the use of advance care planning over time in all settings could represent better integration of care into practice. Those who died of acute respiratory conditions (including flu and pneumonia) may have been less likely to have an advance care plan record due to a shorter illness before death.

### Strengths and limitations

Our study assessed a range of measures and developed two novel indicators of care quality at the end of life, which build on evidence of aspects of care that matter to patients, in keeping with the drive towards patient-centred care,^24^ and opportunities to use routine electronic health records to understand quality of care. The trends in use of these measures, as well as an existing measure of recognition of palliative care, provide new insights into the quality of care in community settings at the end of life at a time of significant change in health services and patterns of end-of-life care.^7–9^ The results are drawn from a large-scale, nationally representative dataset covering 40% of deaths in England, making the scale of analysis unprecedented. The study also uses NHS standard coding—SNOMED—and the analysis code is openly available for reuse. The advisory group included patients in receipt of end-of-life care as well as family carers, general practitioners and academic and policy experts, who contributed to the design of the study and interpretation of the data.

The indicators do not reveal the day-to-day experience of care. For instance, while the presence of an advanced care plan is positive, the plan needs to be revised and shared appropriately between services and followed to be meaningful. As such, these measures should be treated as distinct from patient-reported outcome measures. Being reliant on GP data limited our access to detailed information and documentation of end-of-life processes. The Electronic Palliative Care Co-ordination Systems,^25^ a dataset designed for documenting palliative care activity at a regional level, may make a better resource for understanding palliative care activity with a more granular lens if these data were incorporated into linked datasets.

The indicators developed in this study rely heavily on clinical coding practice and may not necessarily represent care quality. The analysis is therefore vulnerable to coding errors, and further research should consider implementing a sensitivity analysis to understand the potential impact of coding errors on each indicator. Additionally, trends over time were not confirmed statistically, and findings remain reliant on face validity. Further research could strengthen findings by testing for associations between indicators to confirm internal consistency and remove reliance on face validity.

### Policy implications

We identified changes in coding relating to changes in demand for services and disruption to usual services at the start of the COVID-19 pandemic (March 2020), most notable for advance care planning and palliative care measures. For the palliative care measures, we continued to see an impact on recording during the second wave (September 2020–April 2021). Since then, all measures have been gradually increasing for all deaths, with differing trends for different places and causes of death. Continuing to track changes in coding in primary care is critical at a time when there is a significant change in the provision of services, as well as the use of electronic health records.

The palliative care needs and advance care planning measures both show value in understanding trends in end-of-life care, have face validity and could readily be implemented in the NHS and adapted for use in different settings.

Better coding and linkage between GP and other clinical records would result in better estimates of these measures using this methodology. This will ultimately contribute to a wider pool of knowledge on end-of-life care services in England and support national monitoring through the forthcoming Modern Service Framework,^26^ especially vital in a post-pandemic world where the landscape of end-of-life care delivery has changed.

## Conclusions

This work pilots the use of electronic health records data, accessed via OpenSAFELY, to construct measures of quality of end-of-life care that are meaningful to patients and relevant to end-of-life care delivered in community settings. Two new measures were produced, specialist palliative care team contacts and advance care planning, and their use over time by cause and place of death was tracked. Advance care planning was more likely to accurately reflect care activity, as opposed to provider coding practices, rendering it a useful measure.

## Supplementary material

10.1136/spcare-2025-005695 online supplemental file 1

10.1136/spcare-2025-005695 online supplemental file 2

10.1136/spcare-2025-005695 online supplemental file 3

## Data Availability

All data relevant to the study are included in the article or uploaded as supplementary information.
