# Supplementary material for: Electronic health record data to develop indicators of end-of-life care quality
Source: BMJ Support Palliat Care. 2026 Apr 14;16(6):e005695. doi: 10.1136/spcare-2025-005695 (PMC13725638; doi:10.1136/spcare-2025-005695)
Supplement: Supplementary file 1 [file spcare-16-6-s001.pdf]

Supplemental 1:

Codelist for specialist palliative care team contacts:

code	term
1078411000000101	Referral to community specialist palliative care team
1083441000000100	Signposting to palliative care team
1092551000000109	Under care of hospice at home service
1148679005	Specialist palliative care service
193111000000103	Provision of palliative care in own home
193121000000109	Provision of palliative care in hospital, hospice or care home
201511000000105	Under care of palliative care service
246931000000107	Under the care of community palliative care team
251481000000106	Under the care of community palliative care team
25251000000109	Discharged from community specialist palliative care team
25411000000109	Referred to community specialist palliative care team
25761000087103	Inpatient palliative care environment
274461000000103	Under the care of community palliative care team
305284002	Admission by palliative care physician
305381007	Admission to palliative care department
305496007	Under care of palliative care physician
305686008	Seen by palliative care physician
305824005	Seen by palliative care medicine service
305981001	Referral by palliative care physician
306237005	Referral to palliative care service
306288008	Referral to palliative care physician
306442004	Discharge by palliative care physician
306597006	Discharge from palliative care service
309939001	Palliative care department
310073009	Palliative care service
36611000000103	Referred to community specialist palliative care team

36621000000109	Referred to community specialist palliative care team
366809002	Referral to palliative care service
366889004	Referral to palliative care service
36871000000109	Discharged from community specialist palliative care team
36881000000106	Discharged from community specialist palliative care team
381841000000101	Seen by palliative care service
381851000000103	Seen by palliative care service
381861000000100	Seen by palliative care service
393100003	Community specialist palliative care
393102006	Specialist palliative care
394051004	Community specialist palliative care
394053001	Specialist palliative care
395086005	Community specialist palliative care
395092004	Specialist palliative care
395586002	Specialist palliative care treatment - inpatient
395587006	Specialist palliative care treatment - daycare
395588001	Specialist palliative care treatment - outpatient
395631008	Specialist palliative care treatment - inpatient
395632001	Specialist palliative care treatment - daycare
395633006	Specialist palliative care treatment - outpatient
441874000	Seen by palliative care service
818361000000102	Discharge from community specialist palliative care
850951000000107	Under care of palliative care specialist nurse
850961000000105	Under care of specialist palliative care nurse
874891000000101	Under care of palliative care physician

Codelist for advance care planning:

1024591000000100	Review of emergency health care plan
1024601000000107	Emergency health care plan reviewed
1025611000000105	End of life care plan offered
1025621000000104	End of life care plan offered
1033761000000101	Has end of life care plan
1046711000000106	Emergency health care planning
1050581000000105	Review of end of life advance care plan
1050701000000108	Has end of life care plan
1064391000000100	Multi-agency action plan
1064401000000102	Vulnerable adult care plan
1089601000000103	Resuscitation plan
1089611000000101	Paediatric resuscitation plan
109011000000104	Review of care plan
1092351000000103	Care Programme Approach care plan
1092361000000100	Care Programme Approach care planning
1093971000000100	Prevention of delirium care plan
1095081000000100	End of life advance care plan
1095101000000106	Offer of dementia advance care planning review declined
1095111000000108	Review of dementia advance care plan
1095121000000102	Dementia advance care plan agreed
1095131000000100	Offer of end of life advance care plan review
1095151000000107	Has end of life advance care plan
1095161000000105	End of life advance care plan review declined
1095591000000106	Surgical care plan
1095851000000100	Advance care plan agreed
1103771000000105	Advance care planning review offered
1107891000000106	Has Recommended Summary Plan for Emergency Care and Treatment
1137426009	Able to participate in care planning

1148161000000104	Review of dementia care plan
1148840002	Family able to participate in care planning
1187911000000105	Personalised Care and Support Plan agreed
1187921000000104	Review of Personalised Care and Support Plan
1228801000000107	Transplant surgery care plan
1256111004	Coordination of care of subject with caregiver
1280001000000105	Thoracic surgery care plan
1290241000000100	Respiratory physiology care plan
1290251000000102	Renal medicine care plan
1310771000000105	Rare disease care plan
1331271000000102	Stroke medicine care plan
133891005	Care planning session
1362301000000101	Acute internal medicine care plan
1362351000000100	Emergency medicine care plan
1362441000000109	Endocrine surgery care plan
1362481000000101	Medical oncology care plan
1362791000000109	Vascular physiology care plan
1363591000000107	Neurology care plan
1363611000000104	Neurosurgical care plan
1363621000000105	Nuclear medicine care plan
1363641000000103	Oral and maxillofacial surgery care plan
1363651000000100	Orthogeriatric medicine care plan
1364041000000106	Paediatric palliative medicine care plan
1364051000000109	Palliative medicine care plan
1364521000000106	Trigger factor to activate care plan
1403201000000108	Transient ischaemic attack care plan
1465221000000102	Future care plan
163151000000101	Care plans - care record element
185551000000106	Single assessment process summary care plan completed

188091000000103	Agreement of care plan
188851000000100	Single assessment process summary care plan completed
191591000000101	Agreement of care plan
192351000000107	Single assessment process summary care plan completed
192961000000106	Single Assessment Process care plan
196121000000104	Palliative care plan review
202251000000104	Heart failure care plan discussed with patient
208091000000104	Heart failure care plan discussed with patient
208101000000107	Heart failure care plan discussed with patient
217701000000105	Palliative care plan review
217711000000107	Palliative care plan review
278448002	Care plan
280151000000107	Carer holds patient care plan
280161000000105	Carer holds patient care plan
280171000000103	Carer holds patient care plan
288832002	Care Program Approach care plan
288833007	Care Program Approach care planning
288834001	Agreeing on care plan
288835000	Agreeing on entire care plan
288836004	Agreeing on elements of the care plan
288837008	Care planning under section 117 of Mental Health Act 1983
314705003	Treatment plan given
325661000000106	Care plan
325671000000104	Integrated care plan
325681000000102	Crisis plan
325691000000100	Contingency plan
335021000000109	Care plan review due
335031000000106	Care plan reviewed
335041000000102	Does not agree with care plan

372991000000103	Falls prevention plan
373001000000104	Falls care plan
373011000000102	Falls care plan
373511000000107	Contributes to own support plan - Common Assessment Framework
373521000000101	Manages own support plan - Common Assessment Framework
374421000000100	Thinking ahead gold standard advanced care plan discussion statement
374431000000103	Thinking ahead advanced care plan discussion statement
374441000000107	Thinking ahead advanced care plan discussion statement
376531000000105	Coordinated support plan
393138004	Care plan
394089009	Care plan
397608007	Finding related to care planning
399414003	Multidisciplinary care conference for cancer care planning
406140001	Discussion about care plan with family
413095006	Clinical management plan review
425268008	Review of care plan
492321000000103	Notification to primary care out of hours service of palliative care plan in place
492331000000101	Notification to primary care out of hours service of palliative care plan
492341000000105	Notification to primary care out of hours service of palliative care plan
513391000000101	Vulnerable adult care plan
513401000000103	Vulnerable adult care plan
514501000000101	Agreeing on proposed care outcomes
516281000000102	Patient held care plan
516291000000100	Patient held care plan
523031000000107	Review of personal care plan
523041000000103	Review of personal care plan
523051000000100	Personal care plan completed
523061000000102	Personal care plan completed

523071000000109	Personal care plan offered
525711000000101	Offer of personal care plan accepted
525721000000107	Personal care plan accepted
526611000000100	Has end of life advance care plan
526621000000106	Has end of life care advanced care pathway
60631000210103	Breast cancer multidisciplinary meeting
700225008	Caregiver understands care plan
702779007	Emergency health care plan agreed
711069006	Coordination of care plan
711121005	Facilitation of family ability to participate in care plan
712431000000100	Health and social care plan agreed
712441000000109	Health and social care care plan agreed
712744002	Evaluation of care plan
713058002	End of life care planning
713580008	Review of advance care plan
713581007	Review of advance care plan offered
713598007	Review of anticipatory care plan
713600001	Agreement on advance care plan
713602009	Discussion about end of life care planning
713603004	Advance care planning
713604005	Education about advance care planning
713662007	Discussion about advance care planning
713665009	Discussion about advance care planning with family member
713673000	Has end of life care plan
713987003	Ability to agree with care plan
714361002	Discussion about advance care planning with caregiver
714411005	Able to agree with care plan
714747005	Discussion about advance care planning declined
714748000	Has advance care plan

714831004	Does agree with care plan
715016002	Advance care planning request by patient
715072008	Sharing of advance care plan with out of hours service
716048005	Review of advance care plan declined
718637005	End of life care planning offered
719962002	Advance care planning decisions shared with out of hours service
722504006	Care plan goal agreed
734163000	Care plan
735324008	Treatment escalation plan
735330008	Vulnerable adult care plan
735984001	Heart failure self management plan
736054002	Pressure injury care plan
736057009	Respiratory disorder inpatient care plan
736161001	Education for care planning
736249004	Radiotherapy care plan
736250004	Respite care plan
736251000	Stroke care plan
736283006	Chronic obstructive pulmonary disease clinical management plan
736284000	Diabetes clinical management plan
736352009	Cardiac surgery inpatient care plan
736365000	Acute exacerbation of chronic obstructive pulmonary disease care plan
736366004	Advance care plan
736367008	Anticipatory care plan
736368003	Coronary heart disease care plan
736371006	Dementia care plan
736372004	Discharge care plan
736373009	End of life care plan
736396009	Interventional cardiology inpatient care plan
736400009	Cardiology inpatient care plan

736401008	Chemotherapy care plan
736402001	Hip fracture inpatient care plan
737430008	Congestive heart failure clinical management plan
737431007	Coronary heart disease risk clinical management plan
737432000	Hyperglycemia clinical management plan
737433005	Hypoglycemia clinical management plan
737436002	Hypoglycemia self management plan
737437006	Hyperglycemia self management plan
737438001	Myocardial infarction clinical management plan
744980002	Medical critical care plan
761621000000108	Contributes to own support plan
762101000000102	Manages own support plan
770576004	Prevention of delirium care plan
771082000	Acute medicine care plan
772831000000107	Discussion about advance care plan
773130005	Nursing care plan
773435006	Surgical care plan
773436007	Plastic surgery care plan
773437003	Trauma surgery care plan
773438008	Oral surgery care plan
773441004	Hand surgery care plan
773444007	Pancreatic surgery care plan
773508000	Interventional radiology care plan
773595007	Pulmonary medicine care plan
773596008	Care of elderly care plan
773978009	Endocrinology care plan
773981004	Palliative care plan
774171000000108	Inpatient care plan
774181000000105	Major surgery inpatient care plan

774201000000109	Unplanned inpatient care plan
780681000000100	Review of care plan
781151000000109	End of life advance care plan
783631000000109	General practitioner out of hours service notified of chronic obstructive pulmonary disease care plan
783641000000100	General practitioner out of hours service notified of chronic obstructive disease care plan
785051000000105	Provision of copy of care plan
788031000000109	Gold standards framework advance care plan discussion statement
788041000000100	Gold standards framework advance care plan discussion statement
792871000000101	Has anticipatory care plan
792881000000104	Has anticipatory care plan
793911000000108	Coronary heart disease care plan
793921000000102	Coronary heart disease care plan
795951000000108	Education for care planning
799181000000101	Critical care medical care plan
799191000000104	Critical care medical care plan for major complex surgery
799201000000102	Critical care medical care plan for major surgery
799281000000107	Critical care medical care plan for surgery
79985009	Nursing care planning session
802321000000106	Care plan agreed
802431000000101	End of life care assessment
805381000000101	Offer of end of life advance care plan review
805401000000101	End of life advance care plan review declined
805571000000105	Request for end of life advance care plan
806501000000107	Review of end of life advance care plan
810951000000101	Chronic obstructive pulmonary disease self-management plan review
810971000000105	Heart failure self-management plan review
810991000000109	Stroke self-management plan review

811701000000105 Angina self-management plan review

811871000000107 Health and social care plan agreed

816281000000101 Has advance statement (Mental Capacity Act 2005)

816301000000100 Has advance decision to refuse treatment (Mental Capacity Act 2005)

816321000000109 Has advance decision to refuse life sustaining treatment (Mental Capacity Act 2005)

818661000000108 Education for care planning

818671000000101 Education for care planning

820121000000100 Discharge care plan

820621000000107 Has involved healthcare professional in discussion about advance decision to refuse treatment (Mental Capacity Act 2005)

823581000000101 Multidisciplinary care plan

827011000000106 Has involved healthcare professional in discussion about advance care planning

827021000000100 Has involved healthcare professional in discussion about advance care planning

827571000000106 Has chronic obstructive pulmonary disease care plan

827581000000108 Has chronic obstructive pulmonary disease care plan

834061000000102 Provision of copy of care plan

835181000000106 Agreeing on health and social care plan

837611000000100 Review of supportive care plan

837621000000106 Review of supportive care plan

837651000000101 Personal care plan-based care started

844571000000103 Agreeing on health professional actions in care plan

844581000000101 Agreeing on health professional actions in care plan

844591000000104 Agreeing on patient actions in care plan

844601000000105 Agreeing on patient actions in care plan

846361000000105 Vascular surgery inpatient care plan

846561000000103 Major vascular surgery inpatient care plan

846611000000101 Major urological surgery inpatient care plan

850471000000108 Thoracic surgery inpatient care plan

850501000000101	Cardiac surgery inpatient care plan
851301000000102	Stroke care plan
851311000000100	Respite care plan
851321000000106	Acute exacerbation of chronic obstructive pulmonary disease care plan
851331000000108	Burn inpatient care plan
851341000000104	Cardiology inpatient care plan
851351000000101	Chemotherapy care plan
851411000000109	Respiratory disorder inpatient care plan
851661000000109	Major head and neck surgery inpatient care plan
851671000000102	Hip fracture inpatient care plan
851751000000102	Major orthopaedic surgery inpatient care plan
851781000000108	Neurosurgery inpatient care plan
851791000000105	Major neurosurgery inpatient care plan
851801000000109	Interventional cardiology inpatient care plan
851961000000102	Advance care planning
852221000000107	Major plastic surgery inpatient care plan
852261000000104	Laryngectomy surgery inpatient care plan
854031000000108	Ambulance service notified of patient on end of life care register
860921000000105	Pressure ulcer care plan
861411000000103	End of Life Care Document
861421000000109	End of Life Care Coordination Summary
862221000000100	Surgery inpatient care plan
863581000000107	Provision of written information about advance care planning
863591000000109	Provision of written information about advance care planning
866147001	Care planning for fall risk
866611000000109	Single Assessment Process care planning
869791000000101	Dementia care plan
869801000000102	Dementia care plan
869811000000100	Agreeing on care plan with legitimate patient representative

869821000000106 Agreeing on care plan with legitimate patient representative

872361000000105 Discussion about heart failure care plan

879781000000106 Single Assessment Process care plan

884441000000103 Continence treatment plan given

884451000000100 Continence treatment plan agreed

885791000000102 Emergency health care plan agreed

885801000000103 EHCP (emergency health care plan) agreed

887701000000100 Emergency health care plan

887711000000103 Emergency health care plan

889231000000107 Carer understands care plan

889241000000103 Carer does not understand care plan

889251000000100 Carer demonstrates deficient understanding of care plan

891681000000109 Carer understands care plan

892021000000107 Has Advance Decision to Refuse Treatment (Mental Capacity Act 2005) – Refuses all blood transfusion and administration of primary blood components and minor fractions

905161000000101 Subject actions in care plan agreed

905181000000105 Healthcare professional actions in care plan agreed

910451000000100 Notification to primary care out of hours service of end of life care plan in place

912781000000104 Dementia advance care plan agreed

912791000000102 Advance care plan agreed

912801000000103 Review of dementia advance care plan

912811000000101 Dementia advance care plan review

913631000000104 Admission avoidance care plan

913681000000100 Admission avoidance care plan agreed

913691000000103 Admission avoidance care plan agreed

913721000000107 Review of admission avoidance care plan

913731000000109 Review of admission avoidance care plan

914841000000105 Anticipatory care plan

917311000000109 Advance care planning

917991000000101	Discussion about advance care plan
92011000000102	Agreement of care programme approach care plan
92041000000101	Copy of care programme approach care plan with carer
92051000000103	Copy of care programme approach care plan with friend
92061000000100	Copy of care programme approach care plan with general practitioner
92081000000109	Copy of care programme approach care plan with neighbour
92101000000103	Copy of care programme approach care plan with relative
921531000000108	Education about advance care planning
921581000000107	Anticipatory care planning
922241000000108	Discussion about advance care planning with family member
922251000000106	Discussion about advance care planning with family
922261000000109	Discussion about advance care planning with carer
922271000000102	Discussion about advance care planning with carer
922301000000104	Sharing advance care planning decisions with out of hours service
922311000000102	Sharing advance care planning decisions with out of hours service
92351000000100	Review of care programme approach care plan
926991000000106	End of life care planning
927761000000104	Discussion about advance care planning
927771000000106	Advance care plan
935371000000102	End of life advance care plan
935381000000100	End of life advance care plan
935911000000108	Has end of life care plan
935921000000102	End of life care plan
936801000000109	Discussion about end of life care plan
936811000000106	Review of advance care plan
936821000000100	Review of anticipatory care plan
936831000000103	Has advance care plan
936861000000108	Offer of advance care planning review
936871000000101	Advance care planning review declined

936881000000104	Request for advance care planning
936901000000101	Advance care plan agreed
939091000000102	Review of integrated care plan
939101000000105	Review of integrated care plan
943001000000107	Safeguarding adults protection plan agreed
956841000000106	Dementia care plan agreed
956851000000109	Dementia care plan agreed
956861000000107	Dementia care plan reviewed
956871000000100	Dementia care plan reviewed
956911000000103	Dementia care plan review declined
959361000000105	Dementia advance care plan
959371000000103	Dementia advance care plan
959461000000102	Dementia advance care plan review declined
959471000000109	Dementia advance care plan review declined
959801000000104	Acute medicine care plan
959831000000105	Adult intensive care care plan
959871000000107	Anticoagulation care plan
960041000000107	Cardiothoracic surgery care plan
960051000000105	Cardiothoracic transplantation care plan
960061000000108	Care of the elderly care plan
960791000000104	Respiratory medicine care plan
960851000000104	Specialist multidisciplinary care plan
960861000000101	Specialist palliative care plan
960911000000105	Spinal injury care plan
960921000000104	Spinal surgery care plan
960981000000103	Surgical dentistry care plan
961001000000102	Surgical oncology care plan
961021000000106	Swallow clinic care plan
961081000000107	Trauma and orthopaedics care plan

961621000000103	Palliative care plan
961651000000108	Community palliative care plan
961891000000104	Dementia assessment care plan
962041000000106	Old age psychiatry care plan
962161000000108	General medical care plan
962171000000101	General surgical care plan
962301000000106	Hospice at home care plan
962321000000102	Hospice care plan
962331000000100	Intermediate care plan
962341000000109	Interventional radiology care plan
965811000000106	Admission avoidance care plan review declined
965821000000100	Admission avoidance care plan review declined
966051000000108	Discussion about emergency health care plan
966061000000106	Discussion about emergency health care plan
966381000000100	Review of anticipatory care plan
966391000000103	Review of anticipatory care plan
966931000000100	Treatment Escalation Plan
966941000000109	Treatment Escalation Plan
983861000000106	Discharge care plan
